# Exploring experiences in peer mentoring as a strategy for capacity building in sexual reproductive health and HIV service integration in Kenya

**DOI:** 10.1186/1472-6963-14-98

**Published:** 2014-03-01

**Authors:** Charity Ndwiga, Timothy Abuya, Richard Mutemwa, James Kelly Kimani, Manuela Colombini, Susannah Mayhew, Averie Baird, Ruth Wayua Muia, Jackline Kivunaga, Charlotte E Warren

**Affiliations:** 1 Population Council Nairobi, Nairobi, Kenya; 2 London School of Hygiene and Tropical Medicine, London, UK; 3 Ministry of Health, Nairobi, Kenya

**Keywords:** Mentoring, Integration, HIV, Sexual reproductive health, Postnatal care, Family planning

## Abstract

**Background:**

The Integra Initiative designed, tested, and adapted protocols for peer mentorship in order to improve service providers’ skills, knowledge, and capacity to provide quality integrated HIV and sexual and reproductive health (SRH) services. This paper describes providers’ experiences in mentoring as a method of capacity building. Service providers who were skilled in the provision of FP or PNC services were selected to undergo a mentorship training program and to subsequently build the capacity of their peers in SRH-HIV integration.

**Methods:**

A qualitative assessment was conducted to assess provider experiences and perceptions about peer mentoring. In-depth interviews were conducted with twelve mentors and twenty-three mentees who were trained in SRH and HIV integration. Interviews were recorded, transcribed, and imported to NVivo 9 for analysis. Thematic analysis methods were used to develop a coding framework from the research questions and other emerging themes.

**Results:**

Mentorship was perceived as a feasible and acceptable method of training among mentors and mentees. Both mentors and mentees agreed that the success of peer mentoring largely depended on cordial relationship and consensus to work together to achieve a specific set of skills. Mentees reported improved knowledge, skills, self-confidence, and team work in delivering integrated SRH and HIV services as benefits associated with mentoring. They also associated mentoring with an increase in the range of services available and the number of clients seeking those services. Successful mentorship was conditional upon facility management support, sufficient supplies and commodities, a positive work environment, and mentors selection.

**Conclusion:**

Mentoring was perceived by both mentors and mentees as a sustainable method for capacity building, which increased providers’ ability to offer a wide range of and improved access to integrated SRH and HIV services.

## Background

Integrating sexual and reproductive health (SRH) and HIV services has the potential to increase service utilization, improve quality of services and efficient use of resources, and enable health systems to respond to client needs, leading to improved client satisfaction [1-5]. However, knowledge gaps among frontline providers constrain service provision necessitating training on additional knowledge and skills for SRH and HIV integration [6]. Traditional approaches to capacity building, such as offsite training workshops, are costly, interrupt service delivery, are not conducive to the sharing of newly acquired skills and knowledge between colleagues, and, therefore, have limited sustainability [7-9]. Training in low resource settings is further complicated by a crisis in human resources for service provision [7-10], hence the need for innovative approaches to improving provider skills without compromising service delivery. Mentorship is one such approach, which harnesses the potential of service providers and is likely to enhance the quality of service provision in integrated service settings.

While there is no universal consensus on the definition of mentorship [11], mentoring is an interactive, facilitative process meant to promote learning and development [11,12]. Mentorship can either be formal (generally designed for a predetermined length of time) or informal (based on good rapport and mutual attraction, which tends to develop slowly) [13]. It occurs when a more skilled or experienced person is paired with a less skilled person with the agreed-upon goal of having the less skilled person develop specific abilities to reach long-term objectives [11]. Workplace mentorship has been associated with a wide-range of positive outcomes [14], including increased confidence and self-esteem among mentored individuals compared to their non-mentored counterparts [15]. Mentees also gain more knowledge, experience less stress and conflict, are more satisfied with their jobs, and are less likely to leave their organizations compared to non-mentees [16].

In Kenya, the Integra Initiative aims to strengthen the evidence base on the impact of integrating family planning (FP), postpartum/postnatal care (PNC) and HIV services in sub-Saharan Africa through assessing two models of integration: 1) integration of FP and HIV services referred here as FP model and includes HIV testing, STI screening and management, cervical cancer screening, condom promotion within FP consultations, and referral for HIV services; and 2) The integration of postnatal care (PNC) and HIV services referred as PNC model and includes the provision of postnatal services to mother and baby, postpartum FP services, HIV testing for mother and infant, and referrals for HIV and other services.

Across the two models, one component of the intervention was to build capacity of health workers to provide integrated SRH /HIV services. In collaboration with the Ministries of Health Kenya the Integra Initiative developed mentorship training package. Although studies have shown that mentoring improves provider skills and attitudes [14,17], there is little evidence on the process and dynamics of implementing a mentoring program to build capacity for SRH and HIV integration. This paper explores the experiences of mentors and mentees from selected health facilities in Kenya where SRH-HIV service integration is ongoing. The paper addresses the following research question "how does mentoring work in SRH and HIV service integration? We describe the process, enabling factors, benefits, and challenges of peer mentoring in resource poor settings.

### The mentoring process

**Figure 1:**
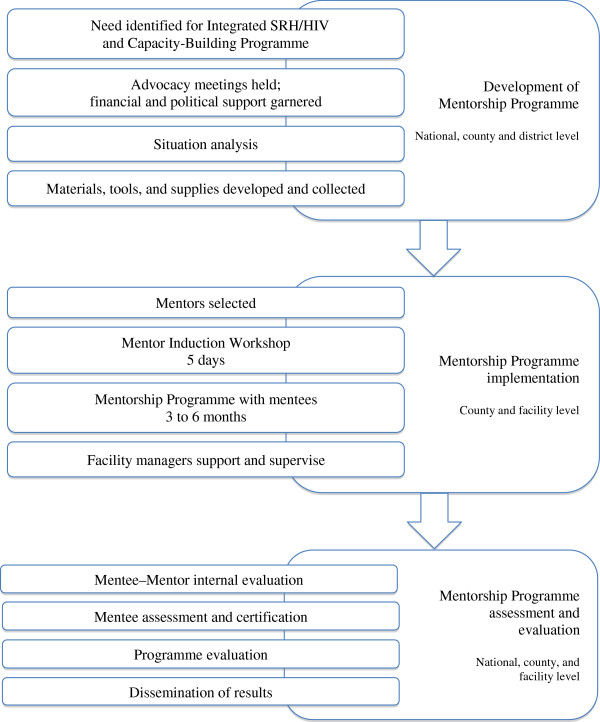
Mentorship Programme Implementation.

Within the Integra Initiative, the mentoring process reported in this paper is based on our reflections within the context of the research and its evaluation conducted within the study. The study jointly with Ministry of Health, Kenya developed a framework as shown in Figure 1 that followed three key steps- 1) development of the mentorship program, 2) implementation of the mentorship program and 3) assessment and evaluation. Each of these steps comprised sub-activities as such advocacy for mentoring among stakeholders; conducting a health facility assessment (situational analysis); development and adaptation of mentoring tools and materials; selection and induction of mentors; conducting peer mentoring sessions in FP, PNC, and HIV service settings; monitoring and supervision of mentoring; and assessment and certification of mentees. These steps were important for standardizing the mentoring approach as training model.

Mentorship activities were implemented between August 2009 and July 2010. Prior to the intervention, the Integra Initiative met with providers, health facility, district, and regional health managers, SRH and HIV trainers, program managers, and policymakers to obtain consensus on the use of mentoring as a training method. Simultaneously, a health facility assessment was conducted; this included an inventory to identify the availability of equipment and supplies; an evaluation of providers’ knowledge and skills, and observations of client-provider interactions. Guidelines and training materials on HIV, FP, postpartum and newborn care, cancer of the cervix, were reviewed and adapted to develop specific mentoring tools (Consensus was reached on the core knowledge and skills needed to attain competency in the provision of integrated HIV and SRH services. Mentoring tools were initially pre tested over a two-month period in six intervention facilities in both PNC and FP integration models (12 in total). The following tools were then revised and finalized: the mentees initial self-assessment; the mentor’s checklist; the mentee logbook; the mentor’s monthly summary sheet; and a job aid on how to use the mentoring tools (Table 1).

**Table 1:** Description of mentoring tools

**Tool**	**Who/When used/Filled**	**How**	**Point of use**	**Comments**
**BCS Plus Toolkit**	Mentors and mentees during service provision	Use algorithm providing step-by-step guidance to provide integrated SRH-HIV services during consultation, refer to trainee guides on how to implement material in the toolkit	In clinical area (FP, PNC, ART, MCH, VCT) or during training sessions	Contains a series of illustrative counselling cards covering a range of topics, such as types of FP methods, STI and HIV risk assessment, and HIV counselling and testing
**Mentees initial assessment pre-test**	Mentee	Answers questions and returns responses to the mentor	In clinical area (FP, PNC, ART, MCH, VCT)	The mentor gives each mentee to assess his/her level of knowledge before starting mentoring
**Mentees clinical protocol**	Mentor when mentoring/coaching the mentee on clinical procedures	Observes mentee performance during clinical procedures in the protocol and scores according to the key provided in the integrated SRH/HIV clinical assessment tool	In clinical area (FP, PNC, ART, MCH, VCT)	The mentor maintains a facility file for all procedures observed
**Mentor logbook**	Mentee after client visit	Summarizes skills acquired after every clinical procedure	In clinical area (FP, PNC, ART, MCH, VCT) or in offices	Maintained by mentees and is availed to supervisors as required
**Monthly summary sheet**	Mentor at end of the month	Records of all mentoring sessions with her/his mentee(s) and summarizes all activities	In clinical area (FP, PNC, ART, MCH, VCT) or in offices	Original copy sent to RH coordinator and one copy kept on file
**Integrated SRH/HIV clinical assessment tool**	Mentor or regional/national level RH coordinator during mentee assessments	Used to evaluate and mark all procedures performed during mentee assessment, a score of 85% means the mentee has passed and can be certified	In clinical area (FP, PNC, ART, MCH, VCT)	A copy is sent to the provincial/national level as evidence of the exam
**Mentees guide**	Mentees for continuous reference	Reference material	Any instance/location when making reference to course content	Maintained by mentees
**Mentees guide**	Mentors for continuous	Reference material	Any instance/location when making	Maintained by mentors

Eligibility criteria for mentor selection included experience in providing HIV and FP or PNC services, the ability to transfer knowledge and skills through behaviour modelling, and a willingness to train others. Mentors from the FP-HIV model had prior training in FP and the use of the Balanced Counselling Strategy (BCS) Plus toolkit for family planning counselling. BCS Plus toolkit provides counseling information on SRH and HIV services and step-by-step guidance for providers on how to use the toolkit during consultations [18]. The mentors participated in a five-day induction workshop, which focused on skills standardization in FP and HIV service provision. For the PNC-HIV integration model, an extra five-day workshop on targeted PNC and the use of BCS Plus was followed by a five-day induction workshop on PNC and HIV service provision.

Each mentor identified a mentee and administered a self- assessment tool to determine his or her individual learning needs. Based on the results, both the mentor and the mentee set learning objectives and agreed on a suitable number of contact hours, which usually took place in the afternoon when facility workloads typically decreased. On average the mentors and the mentees had 100 contact hours over a 4 to 6 months period to achieve the recommend SRH and HIV knowledge and skills competency. After the completion of theoretical trainings, skills demonstrations, return demonstrations and coaching for specific RH and HIV skill sessions were held for mentees. These were followed by clinical practical sessions on clients.

To monitor the effective transfer of knowledge and skills, mentors were observed by supervisors during mentoring sessions. Supervisors conducted verbal question and answer sessions for mentees during site visits and provided knowledge and skill update sessions to both mentors and mentees when necessary. Mentees conducted self and peer skills assessments on a day-to-day basis using learning guides and checklist, and used logbooks to record which competencies were completed satisfactorily. Once mentors were satisfied that mentees had acquired the recommended competencies, mentees were assessed by an external team.

## Methods

This qualitative study was nested within a larger quasi-experimental evaluation of the Integra Initiative- described elsewhere [19]. The Integra Initiative aims to strengthen evidence on the impact of integrating SRH and HIV services in Kenya. Twelve facilities for each integration model (FP or PNC) were purposefully selected to participate based on: client loads of more than 50 per month; facilities with a minimum of two health workers providing FP services; providing a range of SRH services; and not having currently integrated services.

A team of trained researchers conducted in-depth interviews (IDIs) with 23 mentors and 12 mentees providing either FP-HIV or PNC-HIV integration models. We employed purposive sampling to select the study participants. Mentors that had successfully trained at least one service provider using mentoring a methodology for capacity building were included in the study. Mentees that had been trained and certified as competent in RH and HIV services integration through mentoring were included in the study. Mentors and mentees on duty on the day of the interview and consenting to the study were interviewed. Pre-tested interview guides focused on providers’ understanding of the mentoring approach as a method of training, the procedures used in mentoring sessions, the benefits of mentoring in SRH and HIV integration, and challenges faced with the mentoring methodology.

IDIs were conducted in English and lasted approximately 40 minutes. Written informed consent was obtained from all respondents in the study. Interviews were recorded, transcribed in Microsoft Word, and imported to NVivo 9 software (QSR international, 2007) for management and analysis. A thematic framework approach was used to develop a coding framework from the research questions and other emerging themes. Data was coded into themes that emerged from the transcripts. An initial thematic framework was derived through an iterative process and more themes were included as more data were examined. Reflexivity was enhanced through consultations with other members of the research team. Finally, analysis charts were developed to further refine the findings. Quotes selected in presenting results are based on their re- occurrence among respondents, importance, relevance and divergence of opinion on the emerging themes.

### Ethical considerations

Ethical and research clearance for the study were granted by the Population Council Institutional Review Board (Approval No. 443 and 444), the Ethics Review Committee of the London School of Hygiene & Tropical Medicine (LSHTM) (approval number 5426) and the Kenya Medical Research Institute (Approval No. SCC/113 and SCC/114). The study is part of a wider research project under the registration number NCT01694862.

## Results

In total, 35 service providers were interviewed of whom twenty-three were mentors and twelve were mentees. The average age of mentors was 40 years and for mentees 36 years. Mentors had worked at their current facility for an average of nine years and mentees for seven years. Most providers were either registered nurses/midwives or enrolled nurses/midwives (Table 2).

**Table 2:** Provider characteristics

	**Mentees (N = 23)**	**Mentors (N = 12)**	**Total**
**Position**	**n**	**n**	**N**
**Enrolled nurse/midwife**	7	5	12
**Registered nurse/midwife**	12	5	17
**BSCN Nurse**	0	1	1
**Clinical officer**	2	0	2
**Other**	2	1	3
**Total**	**23**	**12**	**35**
**Age**	**n**	**n**	**N**
**<=30 yrs**	5	3	8
**31-35 yrs**	3	4	7
**36-40 yrs**	1	1	2
**41-45 yrs**	6	3	9
**46-50 yrs**	6	0	6
**> 50 yrs**	2	1	3
**Total**	**23**	**12**	**35**
**Time in facility**	**n**	**n**	**N**
**<1 yr**	5	4	9
**1-5 yrs**	4	2	6
**6-10 yrs**	6	2	8
**11-15 yrs**	0	1	1
**16-20 yrs**	0 s	0	0
**> 20 yrs**	5	1	6
**Total**	**20**	**10**	**30**

### Perceptions of the mentoring process

#### Mentors induction

The majority of mentors interviewed felt that they were adequately prepared for the mentorship induction process,

"*Yes, what I would say it [induction] was adequate because I already had knowledge and skills. … the long-term methods, I had the skills, so the update to me was adequate*" [Respondent C04-03, Mentor, FP site].

However, some felt that the five-day induction did not allow for sufficient practice on skills previously trained, but not applied regularly. For instance, one mentor said that the induction period was too short for the IUCD practical sessions.

"…*At first I did not have the right skills and we were taught for only four days so I had to come back to the facility and take a month of practising IUCD insertion before I could mentor somebody"* [Respondent C05-01, Mentor, FP site].

This demonstrates that even if mentors felt that they were fully competent to mentor, in some cases it still took time for them to master and feel confident enough to train others on specific skills. Some mentors required additional support in specific skills, such as screening for cervical cancer lesions using visual inspection with acetic acid/visual inspection with lugols iodine (VIA/VILI). This technique was recently introduced to Kenya [20]. One PNC mentor stated:

"…*even after the training I had to go back to xxxx to practice for a week before I could mentor on VIA/VILI skill*" [Respondent E06-01].

Similarly most mentees from both the FP and PNC sites felt that mentors were adequately prepared to mentor them:

"*My mentor was knowledgeable. When mothers came with complications such as those with IUCDs or those [with] implants so we used to ask her [mentor] what to do and she told us…"* [Respondent C01-05, mentee, FP site].

While another mentee stated

"*he (mentor) was good in demonstrating the use of BCS + toolkit, and although it initially looked hard to build confidence, within a short time I used it with ease"* [Respondent E06-03, Mentee, PNC site].

However, some mentees felt that there were areas where the mentor was not always comfortable such as neonatal care and screening for cervical lesions using VIA/VILI method.

"*…her (mentor) demonstration was not right. I knew it because I had gone for a recent update in neonatal care but I told her (mentor) about the right thing."* [Respondent E05-03, Mentee, PNC site]

A mentee from the family planning sites said

"..*she (mentor) was okay in other areas but for this VIA/VILI…she kept on referring to the Job Aids on the walls and I wondered if we are doing well,…* [Respondent C09-02]

#### Mentees selection

To introduce mentoring, most mentors began with identifying a willing mentee from the same facility. Interviews with mentors indicated that this selection process allowed them to build a relationship of mutual trust, which was conducive for effective learning and development:

"*Now, after the training [induction] I had to choose the mentees.… I had to choose the person who I think would better benefit from my experience… I sold the idea to her first, for her to learn"* [Respondent E01-02, mentor, PNC site]

The majority of the mentees concurred with the above statement and explained they were recruited by asking to volunteer.

*"Our mentor told us to volunteer for the mentoring, so I did since I was interested in learning the skills in long acting FP methods and this was good chance for me".* [Respondent C02-04, mentee, FP site]

### Enabling factors in skills transfer during the mentoring process

#### Trust and mutual respect

Majority of the mentors and mentees reported that mentoring sessions were cordial. As one FP sites mentee described, respect for the mentor was important for the skill transferring.

"*You know although were working together as colleagues, I respected her as one would respect her teacher, I had to humble myself to learn".* [Respondent C01-03, mentee, FP site]

A mentor, PNC site also said*:*

*"I maintained a friendly atmosphere and we worked well as colleagues.... but I had to be firm to ensure that they (mentees) completed their assignments and kept the schedule of our contact time"* [Respondent E02-04]*.*

Most of the mentors thought that factors facilitating the mentorship process included a mentee with an interest in learning and having a complementary working relationship built on trust and mutual respect. As one FP mentor describes the mentorship process*:*

*"You take the pain of doing everything just to make sure this mentee learns......., but mostly it depends on the interest of the mentee"* [Respondent C01-06]*.* Another *Mentor from PNC site* said, "*…My relationship with my mentees… is very good because we have never quarreled at any time* [Respondent E02-04].

Most mentees noted that encouragement from mentors, and patience and cooperation (with both clients and mentors) facilitated the transfer of skills throughout the mentorship process.

"*When the mentor came across a good learning case during her work, she asked the client if I could perform the procedure while a mentor guided me.... most clients agreed’* [Respondent E 04–02, mentee, PNC site].

#### Support and flexibility of managers in mentoring

Some mentors stated that departmental heads supported the mentorship process by providing flexible schedules so that mentees could practice their acquired skills.

"*Although there was shortage of staff, my in charge allowed me to take day shift for six months to avail myself for the mentees’ practical sessions during the day"* [Respondent C01-06, mentor, FP site].

Appreciation for the mentorship and mechanisms for consistent supervision helped to facilitate this higher-level support. For example:

*"The in-charge was supporting us because when you tell her we don’t have Jadelle she could go to district headquarters and borrow and bring to us for learning."* [Respondent E01-02, mentee, PNC site]

In a few instances, the facility managers re-deployed mentees before completion of all the mentoring sessions thus disrupting the training. For example

*"There was a staff change-over, one of the mentee was moved to the operating theater, and it was difficult to continue with training. She and I pleaded with in charge to let her finish but this was not possible, so she took night duty and completed when she was off duty..it was hard for her".* [Respondent 60–01, mentor, from FP sites]

### Benefits of mentoring

#### Individual motivation

Within health facilities, mentoring provided an opportunity for the majority of mentors to be recognized by their managers.

*"I never thought i could train any one, let alone be recognised by the facility in charge and other providers in the district for my work*" [Respondent C01-05, mentor, FP sites].

Majority of the mentees reported that as a result of mentoring the mentees reported that they became motivated to provide quality services to clients and were less likely to refer clients to higher-level facilities since they felt they were more competent with provision of additional skills.

*"The other thing is they appreciate me for what I have done. …They are in a position to come and ask for more services here. Before they used to go to clinicians but nowadays they come to FP clinics if they have problems like STI"* [Respondent C01-01, Mentee, FP site].

Furthermore, they reported offering a wider scope of services. Majority of the mentees reported said that this in return, facilities experienced gains from increased revenue, particularly from long-term FP methods. The increase in the scope of services provided, also saved clients time and money:

"*[Now] we have IUCD clients and before they never used to accept. Also the workload used to be high but now the queues for FP [services] are shorter but we have more clients for VIA/VILI…"* [Respondent C02-05, mentor, FP site]

#### Improved provider-client relationship

The provision of broader integrated HIV and SRH services led to improved relationships between clients and providers:

"*…Because of talking to these mothers, giving them the good services, the long-term methods and screening them for cervical cancer, TB, STI, HIV testing, they have learnt to accept us and they are so kind. They have learnt to love us – not like before when they used to hate us and talk to us in a very rude way"* [Respondent C0-03, mentee, FP site].

Mentoring also improved provider-client communication through improved counselling. This enabled clients to be open about their health issues and service preferences, and often facilitated client diagnosis and improved clients’ understanding of the FP methods available. For example one PNC mentee said:

"*Once you take counselling in a proper way the client is able to expose the deep feelings within her and you are able to offer the services freely because the client will have given her choice on the method."* [Respondent E01- 01]

A mentor also remarked that by offering integrated services providers were now able to help women save some money.

*"…We are saving the mothers the money for transport and method that they had to use every month. They are also appreciating and it has helped to improve the health of our mothers."* [Respondent E05-01, mentor, PNC site]

#### Challenges of mentoring

Competing job priorities and insufficient communication between facility managers and providers demotivated some mentors from initiating mentoring. A FP mentor stated:

"*When I came from the mentors training I explained to the facility in charge and my colleagues what was expected of me. I waited for volunteers so that I could start mentoring, but wacha! (expressing disgusts) they were un bothered,… I only started after the supervisors came."* [Respondent C01-06, mentor, FP site]

Another key challenge that mentors observed was the high workload due to staff shortages. This often limited the contact hours between mentors and mentees, as described by one mentor:

*"Different working schedules and high workload… maybe you plan to have a session on Saturday but it becomes so busy that you miss the time to sit and share or to show a certain mentee a skill."* [Respondent, E01-02, mentor, PNC site]

Almost all the mentors pointed out that their contact time was limited due to a difficulty in coordinating her schedule with that of her mentee:

*"…Now because of the shortage of staff I had a very rough time trying to get time with the mentees because when I am available the mentees are on night duty …so you are getting very few hours for you to sit down with your mentees*" [Respondent C03-04, mentor, FP site].

A few mentors concurred that they did not use the checklist during all the mentoring sessions.

"*sometimes you could get a good case to teach with but the two of you (mentor and mentee) do not have the mentors checklist near at the time, so you just continue......you may miss a few steps but you have taught"* [Respondent E03-01, mentor, PNC site].

In these cases, mentors supervisors took time to correct the mentors during the on-site assessment or/and asked mentors from other facilities to support training in these specific skills if more coaching was required. In cases where mentors were junior in professional rank to a mentee, some mentees with higher ranks expressed discomfort:

"…*She was my junior. She was there training me. But now I had to deal with that… I had to show… I had to make sure that she doesn’t feel like she’s my junior training no, ‘hiyo tuliweka kando’* [we put that aside] *for learning to take place*" [Respondent E01-06, mentee PNC site].

Mentees expressed some concern about the orientation process, which they felt did not provide sufficient information on what would be expected of them:

*"I wish we would have been… told what amount of time was required from us. We were not told the date when we would be assessed. It is good to know when you are starting and for how long so that you can have a time limit* [Respondent E02-01 mentee PNC site].

Some mentees also had difficulty learning less common procedures such as cervical cancer screening, syndromic management of STIs, and IUCD insertions. Mentees described their experiences learning these procedures:

*"I had challenges inserting IUCDs because clients were not used to the method, yet we were required to practice a lot. Screening for cancer of the cervix was also not easy. We only used to hear about [it] so when the mentor trained me, it was not easy…"* [Respondent C01-05, mentee, FP site].

A lack of clients demanding relevant services also hindered mentees’ ability to acquire new skills:

"*…having that patience to wait for that client and wait to be shown… the availability of the clients themselves because if you don’t get a client then it is not easy to learn"* [Respondent E01-02, mentee, PNC site].

Limitations for the mentee arose if mentors did not follow the recommended steps in training mentees for all the skills and techniques included in the mentoring tools as components of SRH and HIV integration. One FP mentee expressed,

*"So we faced great difficulties. I am being assessed with the new techniques and she has taught me with the old techniques* [Respondent C02-04]*."* Another said, "*when we began, we were not following the mentors’ checklist, so when the supervisors asked me for a demonstration of the implants insertion, I missed a few step and I was corrected.... my mentor and I had only used the checklist once"* [Respondent E04-02, mentee, PNC site].

In some instances the mentors and mentee experienced similar challenges. Some mentees said that shortages of commodities affected the amount of training time for mentees and resulted in delayed certification.

"*I had only done three case of Jadelle and then the stock finished, I waited for a month before I could continue with learning that procedure*" [Respondent E05-03, mentee, PNC site]

A FP mentor recalled:

*"We had stock-outs of some of the methods that we were promoting like the Jadelle, Depo, and also the solutions for doing the cervical cancer screening. So when you stay for three weeks without such commodities, even mentoring becomes a problem since you do not have all the things required to work."* [Respondent, C01-05]

Additionally, some also stated that inadequate space for service provision, particularly private rooms for counseling, slowed the process. One PNC mentor explained the situation:

*"We do not have enough rooms. We may train all our staff, but still the rooms remain two. We need privacy. We need room for counseling the client and it is not there*" [Respondent E01-03]. Another PNC mentee stated "our *room has very limited space when we (mentors and mentee), the mother and the baby we have no space but we had just to learn"* [Respondent, E05-03].

## Discussion

The objective of this study was to explore the experiences of mentors and mentees from selected health facilities in Kenya where SRH-HIV service integration is ongoing. The primary aim of introducing mentoring was to strengthen providers’ capacity (knowledge and skills) to provide the recommend package of SRH and HIV integrated services. From the both the mentors and mentees experiences four key components were critical in enabling effective skills transfer: 1) support and flexibility by managers; 2) adequate commodities, supplies, and human resources; 3) mentors selection, induction and use of mentoring tools; and 4) positive work environment. However, both mentors and mentees experienced challenges, which limited the effectiveness of the approach. These challenges included, erratic supplies and commodities, high client case load, shortage of staff, and inadequate skill and/or lack confidence by mentors in training certain skills. Facility-specific solutions to these challenges were identified between mentor and mentees, and the Integra Initiative and/or facility managers offered support as needed.

Selection of mentors is critical in ensuring smooth transfer of knowledge and skills. We found that the majority of the mentors were well selected and thus effective in mentoring. However, there were some cases in which mentors felt they were not sufficiently confident or competent to conduct procedures, such as cervical cancer screening or IUCD insertion. Further, the findings seem to suggest that some mentors did not constantly use the mentoring tools and potentially transferred incorrect knowledge and skills to the mentees. This may have been because the five-day mentor training sessions did not allow for enough practical application of these newly acquired or rarely used skills. In addition, a few of them did not adhere to the mentoring standards. Although, this was addressed during the supervisors visits through onsite training, perhaps there is need to ensure these training gaps are addressed in the course of mentoring. Further, all mentors should adhere to use of mentoring checklist in order to ensure that mentees are assessed on sequence of step in procedures that they may not have been trained on. These findings seem to concur with a study on. use of on job training on post abortion care in Nepal that observed maintaining quality of on-job-training is difficult, particularly if on wide scale [21].

Before initiating a mentoring strategy, it is necessary to have support and endorsement from local facility and high-level managers to ensure a smooth rollout at the facility-level. Mentors and mentees noted that the flexibility of supervisors and managers facilitated the necessary adjustments to their work schedules, allocation of clients and commodities to ensure mentorship on necessary skills, and contributed to a positive learning and teaching environment. World Health Organization (WHO) guidelines on clinical mentoring underscores the need to involve managers at all levels of health care in order to institutionalise mentoring within the public health system [22].

Mentoring was largely reported as feasible and acceptable by both the mentors and the mentees in the study. However, chronic shortages of human resources relative to client loads rendered it difficult for mentors and mentees to coordinate their schedules, which limited contact time. Inadequate infrastructure and critical supplies can also hinder the ability of mentees to acquire new skills. For example, the erratic supply of cervical cancer screening reagents reduced the amount of practice time mentees had to develop and advance their skills and limited the package of integrated services providers were able to offer clients. Peer mentoring as a training strategy is limited by human resources crisis and the ever growing volume of clients that require the services [23]. Where facility managers were committed to implementing the mentorship approach they made deliberate efforts to obtain supplies and commodities – even from nearby facilities if needed – and shortages and stock-outs were limited.

The findings further demonstrate that in a few isolated cases mentoring sessions would reduce manpower (since two providers - mentor and mentee would be with the same clients) and this disrupted services delivery at the facilities, countering the intended effects of the mentoring approach. However, mentoring resulted in improved scope of services as the provider offered a wider range of services after the mentoring process was completed. The findings also demonstrate that mentoring as an approach can be used to improve providers’ technical skills that are one of the major challenges in human resources for health. Mentorship created a sustained mechanism of capacity building for knowledge and skills in SRH/HIV integration despite the low staffing levels. It ensures continuity of services and learning at the same time for the staff and thus can be applicable in low resources settings.

A sense of motivation and willingness to improve skills among mentees and mentors cultivated positive learning environments in facilities. It also developed healthy relationships among providers prospectively leading to increased confidence in performing the skills and commitment to providing quality services. In other studies that examined factors associated with working environment and job retention of health care providers education achieved while on the job was found to be associated with job satisfaction [24-26]. The provision of learning opportunities through mentoring decreases provider anxiety about the future, satisfies career development needs, and creates a high level of job satisfaction [14,27].

Mentoring also encouraged performance feedback, intra-staff communication, and prompted opportunities for facility staff to offer the necessary support. These on-the-job features are critical for enabling service providers to establish a sense of organizational identity and belonging, which increases staff retention [17].

## Conclusion

Overall, most providers perceive peer mentoring to be an effective and feasible approach for capacity building in the context of integrated SRH and HIV services. The benefits of mentoring are particularly relevant for settings with moderate or high HIV prevalence, limited funding for provider capacity building and staff shortages. However, the findings from this qualitative study underscore the need for flexibility and cooperation among peers as well as managers when implementing mentorship.

## Competing interests

The authors declare that they have no competing interests.

## Authors’ contributions

CEW and SM conceived of the study idea and critically reviewed the manuscript. CN, RM and JK developed and pre-tested the in-depth interview guide. CN and TA analysed the data and drafted initial manuscript. JJK, and AB critically reviewed the manuscript. MC and RM critically reviewed and edited the manuscript. CN and RWM coordinated the mentoring implementation activities. JK coordinated data collection fieldwork. All authors read and approved the final manuscript.

## Pre-publication history

The pre-publication history for this paper can be accessed here:

http://www.biomedcentral.com/1472-6963/14/98/prepub
